# A Comparison of Genetic Diversity of COX-III Gene in Lowland Chickens and Tibetan Chickens

**DOI:** 10.1155/2017/8064613

**Published:** 2017-07-03

**Authors:** Xueqin Liu, Pu Zhang, Gongying Zhang, Sichen Li, Long Zhang, Zhongxian Xu, Tianyuan Ma, Diyan Li

**Affiliations:** Institute of Animal Genetics and Breeding, College of Animal Science and Technology, Sichuan Agricultural University, Chengdu, China

## Abstract

To obtain a full understanding of the genetic diversity of the cytochrome oxidase III gene* (COX-III)* and its association with high altitude adaptation in Tibetan chickens, we sequenced* COX-III* in 12 chicken populations (155 Tibetan chickens and 145 other domestic chickens). We identified a total of 11 single nucleotide polymorphisms (SNPs) and 12 haplotypes (Ha1–Ha12). Low genetic diversity (haplotype diversity = 0.531 ± 0.087, nucleotide diversity = 0.00125) was detected for* COX-III*, and haplotype diversity of Tibetan chicken populations (0.750 ± 0.018) was markedly higher than lowland chicken populations (0.570 ± 0.028). Obvious genetic differentiation (nucleotide divergence = 0.092~0.339) and conspicuous gene communication (gene flow = 0.33~32.22) among 12 populations suggested that Tianfu black-bone fowl (white feather) was possibly introduced from Tibetan chicken. SNP m.10587 T>C affects the specific functions of the COX enzyme. Haplotype Ha3 was found in Tibetan chickens, and SNP m.10115G>A caused an amino acid substitution (Val62Ile) associated with phospholipid binding, while mutations m.10017C>A and m.10555G>A and the previously reported SNP m.10065T>C reduced the hydropathy index to some extent. Together, this indicates that the mitochondrial membrane is more hydrophobic in Tibetan chickens.

## 1. Introduction

Domestic chickens fulfill various roles ranging from food and entertainment to religion and ornamentation [1]. Tibetan chicken is a widely distributed aboriginal chicken breed found at altitudes ranging from 2200 to 4100 m; it has adapted well to high altitudes after over the years of living on the plateau [2]. Generally, long-term exposure to hypoxia in animals reduces metabolic activity, retards development, and increases embryo mortality [3]. However, Tibetan chicken has developed an adaptive mechanism to hypoxia, demonstrated by its increased hatchability and survival rate compared with lowland chicken breeds in high altitude areas of Tibet. With its low weight, small size, and strong chest and legs, the appearance and behaviour of the Tibetan chicken resemble those of the Cochin-Chinese red jungle fowl (*Gallus gallus gallus*), making it particularly good at flying and foraging on the plateau alpine region [2–5]. The breed is also important to the resources used to expand the industry in cold areas of high altitude in China.

Avian species living at high altitudes are characterized by the high oxygen affinity of their haemoglobin [6, 7]. Tibetan birds can improve their physiological performance by enhancing their oxygen transport capacity, which has yielded important insights into the genetic basis of adaptation involving haemoglobin as an oxygen carrier [8, 9]. Taking into account the importance of using oxygen more efficiently under hypoxic conditions, information mining for the cellular respiratory chain is a valuable way of understanding the hypoxia response and adaptation mechanisms.

Mitochondrial DNA (mtDNA) sequences are widely used in molecular evolutionary studies. These sequences are useful for estimating times of species and population divergences, comparisons of relative rates of evolution, and phylogenetic inferences within and between vertebrate species [10]. The complete sequence of the chicken mtDNA is 16,775 base pairs and contains 13 protein coding genes, two rRNA genes, and 22 tRNA genes [11]. Cytochrome c oxidase (COX), the terminal enzyme of the mitochondrial respiratory chain, contains 14 protein subunits in mammals, of which three (COX-I, COX-II, and COX-III) are synthesized in the mitochondria [12]. Mitochondrial cytochrome c oxidase subunit I (COX-I) is the central catalytic subunit of cytochrome c oxidase (complex IV), and* COX-I* gene is used as a standard marker for DNA bar coding to enable species identification in animals [3, 13]. The COX-III protein is an important element in regulating the efficiency of proton translocation in cytochrome oxidase over several turnovers [14]. The* COX-III* gene of bar-headed geese contained a nonsynonymous substitution (Trp-116→Arg) that resulted in a major functional change of amino acid class. This mutation was predicted by structural modeling to alter the interaction between COX-III and COX-I, which contributed to adaptation in mitochondrial enzyme kinetics and O2 transport capacity and may finally contribute to the exceptional ability of bar-headed geese to fly at extreme heights [15].

*COX* has been investigated in several biological studies [3, 6, 16, 17], but studies of* COX-III* have rarely been reported in chickens. However,* COX-III* is an important component of the respiratory chain and is very conserved among species. Adaptive changes in COX activity can alter the ATP supply derived from oxidative phosphorylation during hypoxia [9]. The quaternary structure formed by different protein subunits is stabilized mainly through hydrophobic interactions in spite of hydrogen bonding and the van der Waals force is also important. We hypothesized that the stability of the COX holoenzyme three-dimensional structure would increase in line with increases in COX-III protein hydrophobicity. Therefore, in the present study, based on the assumption that cytochrome c oxidase activity is more stable in Tibetan chickens, we analyzed* COX-III* SNPs in 12 chicken populations (five lowland and seven highland populations) to better understand the* COX-III* genetic diversity and to determine the contribution of specific SNPs to high altitude adaptations in Tibetan chickens.

## 2. Materials and Methods

In all experimental populations, 5 populations (Muchuan, Emei, Jiuyuan, Black-Tianfu, and White-Tianfu) belonged to lowland chickens; the other 7 populations (Haiyan, Doilungdêqên, Ganzi, Nyingchi, Diqing, Shannan, and Shigatse) which belonged to Tibetan chickens were collected (Table 1). Blood samples were collected from the wing vein. No bird was slaughtered or unexpectedly injured during sampling. The protocol was approved by the Committee on the Care and Use of Laboratory Animals of the State-Level Animal Experimental Teaching Demonstration Center of Sichuan Agricultural University (Approval ID: Decree number S20160906).

### 2.1. DNA Extraction, Amplification, and Sequencing

We extracted mtDNA by salt extraction method [18]. PCR used the known primer pairs F9797: 5′-ACCAATAATACCATCAATCTCC-3′ and R10830: 5′ CGCTTAGTAGAAAGGATAGTGAG-3′ [19, 20]. PCR amplification was performed in a 50 *µ*l volume with 100–150 ng of genomic DNA, 25 mM MgCl2, 2.5 mM of dNTP mixture, 2 mM each primer, 5 *µ*l of 10x buffer, and 1.25 U LA Taq polymerase (Takara, Dalian, China) under the following conditions: denaturation at 94°C for 5 min, then 35 cycles of 94°C for 30 s, 55°C for 30 s, and 72°C for 60 s, followed by a final extension at 72°C for 7 min [19]. PCR products were verified on 1.5% agarose gels, and specific bands were purified using the TIANgel Midi Purification Kit (Tiangen Biotech, Beijing, China). Purified PCR products were sequenced in both directions using the Big Dye Terminator v. 3.1 Cycle Sequencing Kit (Applied Biosystems, Foster City, CA) on the ABI Prism 3100 DNA sequencer (Applied Biosystems) according to the manufacturer's instructions.

### 2.2. Sequence Data Analysis and Statistical Analysis

Raw sequences were aligned and edited by DNAstar software (DNAstar Inc. Madison, WI, USA). We exported all sequences as an aligned FASTA file. Sequence variations were identified using MEGA 6.0 software [21]. Standard population genetics statistics, including haplotypes and number of haplotypes, haplotype diversity within each group* (Hd)*, nucleotide diversity* (Pi)*, nucleotide divergence* (Dxy)*, net genetic distance* (Da)*, coefficient of differentiation* (Gst)*, and gene flow* (Nm)*, Tajima's *D* value neutral test, were defined using DnaSP V5 software [22], whereas median joining network analysis was performed using program network 4.611 (http://www.fluxus-engineering.com/sharenet.htm). The complete mitochondrial genome sequence of the red jungle fowl was used as the reference sequence (GenBank accession number: NC_001323).

Analysis of molecular variance (AMOVA) was estimated using Arlequin 3.0 software [23]. Bayesian inference was performed as previously described [24]. The optimal model for each data set was estimated by the program Modeltest 3.7 [25]. The program BEAUti v1.5.3 (distributed with BEAST) was used to create the input file to run in BEAST (http://beast.bio.ed.ac.uk/). Samples from the posterior were summarized on the maximum clade credibility tree using the program TreeAnnotator v1.4.8 (distributed with BEAST) and visualized using the program FigTree v1.3.1 (http://tree.bio.ed.ac.uk/software/figtree/). Statistical differences in* COX-III* haplotype frequencies between Tibetan chickens and lowland chickens were analyzed using Fisher's exact test; odds ratios (OR) and 95% confidence intervals (95% CIs) were also calculated. *P* value < 0.05 was taken into account as statistical significance. The bioinformatics platform MitoTool (http://www.mitotool.org/) was used to analyze haplotype distribution frequencies between Tibetan chickens and lowland chickens [26]. *P* < 0.05 were taken to be statistically significant.

Protter [27] was used to annotate and predict protein sequence features of the COX-III protein (http://wlab.ethz.ch/protter/start/). Hydropathy plot of the COX-III protein was predicted using the Tmpred Program [28] (http://www.ch.embnet.org/software/TMPRED_form.html).

## 3. Results

### 3.1. Nucleotide Diversity of* COX-III*

The length of 300* COX-III* sequences was truncated into 784 bp (GenBank accession numbers: NC_001323; no insertion/deletions were detected). We calculated the overall base composition of* COX-III* from the 12 chicken populations using MEGA 6.0. Cytosine (C) was shown to be the rarest nucleotide (16%) with guanine (G) to the most common (32%). The A + T% was around half of* COX-III* (52%).

A total of 11 SNPs (including six singleton sites and five parsimony-informative sites (with no insertions/deletions)) accounted for 1.403% of the total 784 bp* COX-III* sequence from 300 individuals.  Table 2 summarized the number of haplotypes,* Hd*,* Pi*, and other information within each group. The number of variable sites in each population varies from 1 in Diqing and Shigatse to 7 in Haiyan, and the sequences from Doilungdêqên, Ganzi, Nyingchi, Shannan, were 5, 5, 3, and 2, respectively. The highest haplotype diversity was found in Haiyan and Doilungdêqên. While the average number of nucleotide differences in Emei from lowland chickens was greater than others. Taken together, a total of 8 haplotypes were identified in lowland chickens, and the overall haplotype diversity, nucleotide diversity, and average nucleotide differences were 0.570 ± 0.028, 0.00155, and 1.216, respectively. In Tibetan chickens, a total of 9 haplotypes were identified, and the overall haplotype diversity, nucleotide diversity, and average nucleotide differences were 0.750 ± 0.018, 0.0016, and 1.250, respectively. The result showed that the genetic diversity of the Tibetan chicken was noticeably higher than that of lowland chickens. After Tajima's *D* value neutral test, all *P* values were greater than 0.1; therefore, the 12 populations belong to neutral mutations.

### 3.2. Nucleotide Divergence and Net Genetic Distance among Populations

Nucleotide polymorphism among populations can be represented by nucleotide divergence* (Dxy)* and net genetic distance in nucleotides* (Da)*. Within the 12 chicken populations, the average* Dxy* was 0.062% (range 0.092%–0.339%) and the average* Da* was 0.196% (range 0%–0.216%) (Table 3). The largest* Da* (0.216%) was found between Diqing and Jiuyuan black chickens (between Tibetan chickens and lowland chickens), and the smallest* Da* (0%) was observed between Haiyan and Doilungdêqên chickens; the smallest* Dxy* (0.092%) was observed between Black-Tianfu and Muchuan black chickens (within lowland chickens) and the largest* Dxy* (0.339%) was found between Diqing and Emei chickens (between Tibetan chickens and lowland chickens).

To pinpoint the most and least closely related populations, we used net genetic distance in nucleotides* (Da)* to demonstrate that Haiyan and Doilungdêqên are most similar in highland chickens (*Da*  =  0%,* Dxy*  =  0.177%) and that Muchuan black-bone fowl and Tianfu black-bone fowl (black feather) are most similar in lowland chickens (*Da*  =  0.002%,* Dxy*  =  0.092%). Conversely, Tibetan chickens (Diqing) appear more distantly related to Jiuyuan black fowl (*Da*  =  0.216%,* Dxy*  =  0.308%) and Emei black fowl (*Da*  =  0.187%,* Dxy*  =  0.339%).

These findings are conclusive of definite genetic differentiation between different groups of chickens, with the strongest differentiation seen between Tibetan chickens and lowland chickens. Net genetic distances in nucleotides* (Da)* are substantially consistent with the outcome of nucleotide differences* (Dxy)*.

### 3.3. Coefficient of Differentiation (*Gst*) and Gene Flow (*Nm*) between Populations

The coefficient of differentiation* (Gst)* can reveal the extent of gene flow and genetic drift to some extent, while gene flow can uncover possible gene infiltration among populations. In lowland and Tibetan chicken populations, distinct gene exchange (*Gst*  =  0.01,* Nm*  =  25.21;* Gst*  =  0.05,* Nm*  =  4.9) was detected between White-Tianfu (lowland chickens) and Haiyan and Doilungdêqên (Tibetan chickens). However, at the same altitude, obvious genetic differentiation was found in lowland geographical populations (*P* < 0.05) while conspicuous gene communication (*Gst*  =  0.01 * Nm*  =  29.34) was only detected in Muchuan and Black-Tianfu chickens. Obvious genetic differentiation appears to have occurred in Tibetan chickens (Table 4). This indicates that differences in varieties caused genetic differentiation and that the introduction of varieties of different regions led to gene exchange.

### 3.4. Analysis of Molecular Variance

AMOVA showed that the percentage of variation within populations (70.43%) was greater than that between populations (29.57%). Fst value was 0.2957 (^*∗∗*^*P* < 0.01) which implied that the genetic divergence within populations was significant. The results indicate that the twelve geographic populations do not produce largely genetic differentiation, while the genetic diversity in* COX-III* gene mainly comes from within the populations (Table 5).

### 3.5. Sequence Variations in* COX-III* Gene

We detected seven synonymous mutations (m.10081A>G, m.10162G>A, m.10270G>A, m.10336A>G, m.10369G>A, m.10587 T>C, and m.10809C>T) and four nonsynonymous substitutions (m.10017C>A, m.10112 G>A, m.10115G>A, and m.10555G>A) in* COX-III*.  Table 6 shows the observed allele frequencies in each polymorphic site between Tibetan chicken and lowland chicken breeds. After using Pearson chi-square test, we found that three SNPs compared with lowland chickens in COX-III gene (m.10115G>A, m.10270G>A, and 10587 T>C) were significantly different with the allele frequency of 9.7%, 91.0%, and 86.5% (^*∗*^*P* < 0.05) in Tibetan chicken, respectively. Similarly, in lowland chicken, the SNP m.10081A>G was significantly different from Tibetan chicken with the allele frequency of 57.2%. The four SNPs (m.10017C>A and m.10369G>A, m.10555G>A, and m.10809C>T) were only distributed in Tibetan chicken breeds with the allele frequency of only 0.6% and 7.7%, while the other three SNPs (m.10112 G>A, m.10162G>A, and m.10336A>G) were only distributed in lowland chicken with the allele frequency of 0.7%. We consider that the seven SNPs showed nonsignificant difference between Tibetan chicken and lowland chicken breeds (*P* > 0.05).

### 3.6. Median Joining Network of Haplotypes and Phylogenetic Analysis

**Figure 1:**
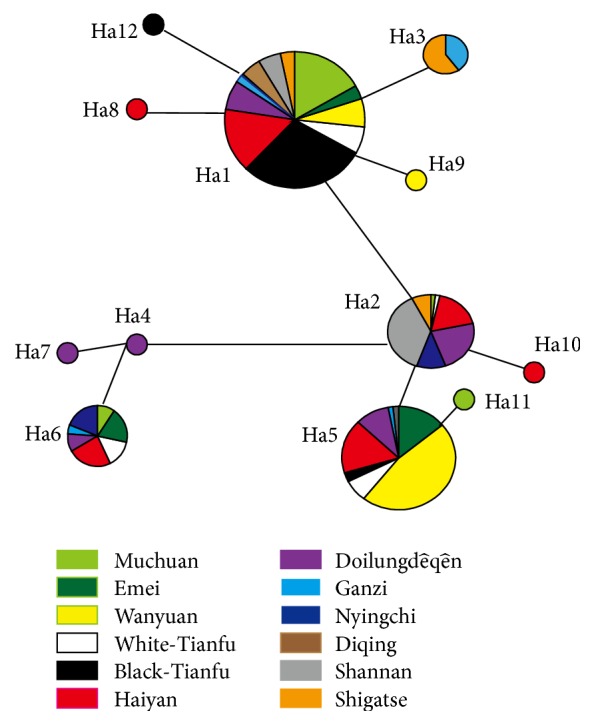
Median joining network of* COX-III* gene haplotypes. Geographic of samples as showed by different colors. Note: population of different regions is replaced by different colors.

We identified 12 haplotypes (Ha1–Ha12) in 300 chickens from the 12 different populations (Table 7). The median joining network was constructed using the 12 haplotypes. Three clusters (A, B, and C) were clearly defined from the network with substantial mutation distances visible between the clusters (Figure 1). Ha1, Ha3, Ha8, Ha9, and Ha12 were restricted to cluster A: Ha1 was the dominant haplotype, present in 87.8% of all individuals in cluster A (130/148 = 87.8%); Ha2, Ha5, Ha10, and Ha11 were restricted to cluster B: Ha5 was the dominant haplotype, present in 55.03% of all individuals in cluster B (71/129 = 55.03%); Ha4, Ha6, and Ha7 were restricted to cluster C: Ha6 was the dominant haplotype, present in 91.3% of all individuals in cluster B (21/23 = 99.3%). The ancestral haplotype was mainly distributed in the center of the median joining network, with derivative haplotypes spreading outwards from it. This indicates that Ha1, Ha2, and Ha4 are the earliest haplotypes.

**Figure 2:**
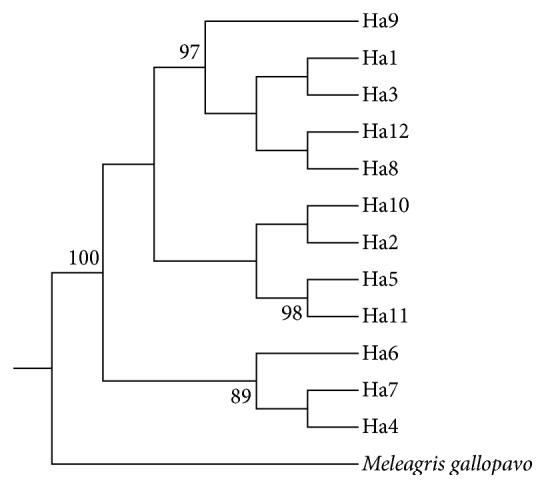
Constructing a phylogenetic tree using the Bayesian method. Note: phylogenetic tree of chicken based on haplotype sequence variation of* COX-III* gene; numbers at the nodes are bootstrap values.

We also used the* Meleagris gallopavo* as an outside group to construct a phylogenetic tree using the Bayesian method. The correlation of the 12 haplotypes is shown in Figure 2, and three clusters can also be seen in the Bayesian tree.

### 3.7. Association between Haplotype Distribution and Altitude Adaptation

Haplotypes of sample sizes under five were not taken into account here. After reviewing the level of significance by Bonferroni correction, haplotype Ha2 was found to be significantly associated with high altitude adaptation at the 0.05 level (*P* value, 1.911 × 10^−14^^*∗*^; OR, 30.375, 95% CI, 7.332–125.837). Haplotypes Ha1 and Ha5 also appear to be significantly associated with lowland adaptation (H1: *P* value, 0.001; OR, 1.918; 95% CI, 1.286–2.860; H5: *P* value, 0.00022, OR, 2.685, CI, 1.567–4.598), whereas haplotype Ha6 does not seem significantly associated with altitude adaptation at the 0.05 level (*P* value, 0.658; OR, 0.74736, 95% CI, 0.310–1.801) (Table 8).

### 3.8. Prediction and Analysis of Secondary Structure Changes in the COX-III Protein

**Figure 3:**
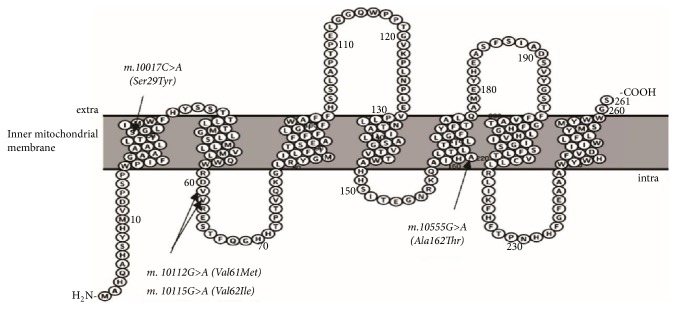
Diagram of transmembrane structure of COX-III protein predicted using the Protter program. The transmembrane structure of the coding sequence was the structure in Cochin-Chinese red jungle fowl. The transmembrane structure of the coding sequence was predicted by nonsynchronous mutations (m.10017C>A m.10112 G>A m.10115G>A m.10555G>A) in the present study.

**Figure 4:**
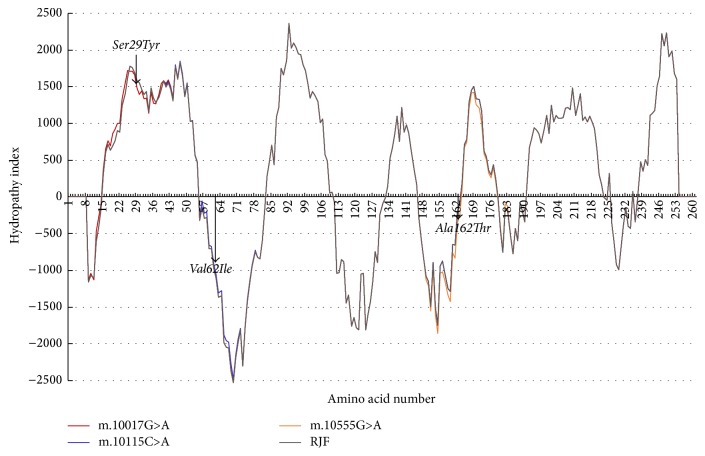
Negative values indicated interaction with water (hydrophilic), and positive values indicated getting out of water (hydrophobic). The red was the hydrophobic index of coding sequence with mutation m.10017G>A; the blue meant the hydrophobic index of coding sequence with mutation m.10115C>A; the orange was the hydrophobic index of coding sequence with mutations m.10555G>A; the irregular curve in gray was the hydrophobic index of coding sequence in the RJF.

Protein sequencing showed that the Tibetan chicken-specific nonsynonymous* COX-III* variants m.10017C>A and m.10555G>A are located in the transmembrane helical structure, while SNP m.10115G>A is located in the outer surfaces of the inner mitochondrial membrane (Figure 3). The hydrophobicity of the COX-III protein was not changed by the Val62Ile amino acid change caused by variant m.10115G>A (Figure 4), but this change at a key site of phospholipid binding may affect the combination of phospholipids related to adaptations to a hypoxic environment. Similarly, nonsynonymous substitutions Ser29Tyr (caused by SNP m. 10017C>A) and Ala162Thr (caused by m. 10555G>A) reduced the hydropathy index to some extent.

## 4. Discussion

Oxygen is one of the critical determinants for normal embryonic and foetal development. In avian embryos, a lack of oxygen causes high foetal mortality, heteroplasia, and cardiovascular dysfunction. The Tibetan chicken breed is native to Tibet and can survive with high hatchability regardless of the negative effects of hypoxia. Animals adapted to high altitudes are characterized by high haemoglobin concentrations and oxygen affinity [6], while lowland chicken breeds suffer polycythemia and ventricular hypertrophy at high altitudes [2, 4, 29].

By analyzing population haplotype diversity and the structure of chicken breeds in southwestern China based on* COX-III* sequences, we found that the Tibetan chicken has a higher level of haplotype diversity (0.750 ± 0.018) than lowland chickens (0.570 ± 0.028). For the results observed, we think that artificial selection leads to reduced nucleotide diversity in lowland chickens. Although 11 SNP sites were identified in* COX-III* from 12 geographic populations, the average genetic distance was only 0.196%, revealing a low level of genomic polymorphisms in all populations. This low level of genetic diversity indicates that this gene is functionally important and hence has an evolutionary constraint. The 12 populations belong to neutral mutations (*P* > 0.1). This suggests that these populations are stable and have not undergone a population expansion in the past few years.

The analysis of the coefficient of differentiation and gene flow between populations suggests that some chicken populations have undergone genetic differentiation at places of equal altitude. This may reflect the introduction of varieties of different regions. For instance, the Tianfu white-bone fowl from a lower altitude has a close population genetic relationship with the Tibetan chicken (*Gst*  =  0.01,* Nm*  =  25.21;* Gst*  =  0.05,* Nm*  =  4.9), indicating that the Tianfu white-bone fowl was introduced from high altitude area. Moreover, Tibetan chickens from Haiyan and Doilungdêqên regions were most similar (*Da*  =  0%,* Dxy*  =  0.177%) suggesting that the genetic distance of the two regions is very close, and gene exchange is very rich (*Gst*  =  0.01,* Nm*  =  32.22).

The regulating mechanism in* COX-III* gene difference is still ambiguous between lowland and highland populations. A previous study detected SNPs in three mitochondrially encoded subunit genes of chicken* COX*, including only one in* COX-III* (m.10081A>G) between an expanded sample of 56 Tibetan chickens and 152 lowland birds [20]. Another study identified [19] eight SNPs, of which five (m.10081A>G, m.10115G>A, m.10270G>A, m.10336A>G, and m.10447C>T) showed significant differences between Tibetan chickens and lowland chickens. Only the synonymous mutation m.10081A>G was found to differ between haplotypes H4 and H5, and chickens with the A allele at m.10081A>G had a probability of being over 2.6 times better adapted to hypoxia than those with the G allele indicating that m.10081A>G may be a prerequisite for shaping high altitude adaptation-specific haplotypes. Our focus on* COX-III* SNPs to explore the different haplotypes detected a novel mutation associated with high altitude adaptations. AMOVA showed that* COX-III* variation mainly existed within a population. Of the 12 defined haplotypes, the existence of Ha4, Ha7, Ha8, and Ha10 only in highland chickens and Ha9, Ha11, and Ha12 only in lowland chickens indicates different degrees of genetic divergence between Tibetan chickens and lowland chickens. Ha1, Ha2, and Ha4 were found to be the earliest ancestors (Figure 1), while haplotype Ha1 was common to all populations suggesting that it is more stable and capable of adapting to new environmental selection. Moreover, Ha2 had significant relationship with high altitude adaptation (*P* value, 1.911 × 10^−14^^*∗*^; OR, 30.375, 95% CI, 7.332–125.837), with the C allele at m.10587 T>C was found to have a probability of being over 30.375 times better adapted to hypoxia than the T allele. We propose that m.10587 T>C affects the functions of the COX enzyme in a similar way to the effect of m.10081A>G [19] on high altitude adaptation of the Tibetan chicken. However, haplotype Ha1 simultaneously contained mutations m.10081A>G and m.10587 T>C and was negatively associated with high altitude adaptation. The function of these mutations warrants need to further research.

Three of the four nonsynonymous mutations identified in the present study (m.10017C>A, m.10115G>A, and m.10555G>A) were peculiar to highland chickens. Mutation m.10115G>A (with the allele frequency of 9.7%, ^*∗*^*P* < 0.05), shared by populations Ganzhi and Diqing, caused the amino acid mutation Val62Ile (Figure 1). A previous study reported SNP m.10115G>A as an uncommon missense mutation in the Tibetan chicken mtDNA genome [30], while another study found that more than one-third of Tibetan chickens (44/125, 35.2%) harboured this mutation, suggesting that it might be associated with high altitude adaptation [19]. In the current study, we found that mutation m.10115G>A is a key site for phospholipid binding, suggesting that it impacts on the function of the mitochondrial membrane in Tibetan chicken. Whereas the other two nonsynonymous mutations (m.10017C>A m.10555G>A) and the reported SNP m.10065T>C [19] both reduced hydropathy index to a certain extent.

## 5. Conclusions

In conclusion, we found largest genetic differentiation between Tibetan and lowland breeds and identified haplotype Ha2 is associated with Tibetan chicken populations. The possible association between increased hydrophobicity/reduced hydrophilic characteristics of the mitochondrial membrane and high altitude adaptation could provide a theoretical reference for poultry genetics. Therefore, our results provide a theoretical basis for future research into fowl breeding.

## Figures and Tables

**Table 1:** Information of the sample used in this study.

Type	Breeds	Population	Altitude	Sample (*n*)	Haplotype distributions (number of birds)
Lowland chicken	Muchuan black-bone	Muchuan	400 m	26	Ha1 (22) H2 (1) Ha6 (2) Ha11 (1)
	Emei black fowl	Emei	500 m	18	Ha1 (4) Ha5 (10) Ha6 (4)
	Jiuyuan black fowl	Jiuyuan	800 m	43	Ha1 (9) Ha5 (33) Ha9 (1)
	Tianfu black-bone fowl (black feather)	Black-Tianfu	800 m	44	Ha1 (38) Ha5 (2) Ha6 (3) Ha12 (1)
	Tianfu black-bone fowl (white feather)	White-Tianfu	800 m	14	Ha1 (8) Ha2 (1) Ha5 (5)

Highland chicken	Tibetan fowl	Haiyan	3100 m	49	Ha1 (20) Ha2 (10) Ha5 (12) Ha6 (5) Ha8 (1) Ha10 (1)
	Tibetan fowl	Doilungdêqên	3658 m	33	Ha1 (9) Ha2 (13) Ha4 (1) Ha5 (7) Ha6 (2) Ha7 (1)
	Tibetan fowl	Ganzi	3390 m	10	Ha1 (2) Ha3 (6) Ha5 (1) Ha6 (1)
	Tibetan fowl	Nyingchi	3100 m	11	Ha1 (1) Ha2 (6) Ha6 (4)
	Tibetan fowl	Diqing	3280 m	15	Ha1 (6) Ha3 (9)
	Tibetan fowl	Shannan	3700 m	29	Ha1 (7) Ha2 (21) Ha5 (1)
	Tibetan fowl	Shigatse	3836 m	8	Ha1 (4) Ha2 (4)

**Table 2:** Nucleotide polymorphism of *COX-III* gene sequence within 12 populations.

	Populations	Number of variable sites	Number of parsimony-informative sites	Number of haplotypes	Haplotype diversity *(Hd)*	Nucleotide diversity *(Pi)*	Average number of nucleotide differences (*K*)	Tajima's *D*
Lowland chicken	Muchuan	5	3	4	0.286 ± 0.112	0.00092	0.720	−1.27664
	Emei	4	4	3	0.627 ± 0.086	0.00209	1.621	1.18115
	Jiuyuan	3	2	3	0.375 ± 0.076	0.00099	0.777	0.25955
	Black-Tianfu	5	4	4	0.255 ± 0.086	0.00079	0.559	−1.27905
	White-Tianfu	2	2	3	0.582 ± 0.092	0.00130	1.022	1.69598
	All lowland chickens	7	4	8	0.570 ± 0.028	0.00155	1.216	−0.07994

Highland chicken	Haiyan	7	4	6	0.727 ± 0.034	0.00199	1.293	0.37142
	Doilungdêqên	5	4	6	0.742 ± 0.043	0.00147	1.152	−0.17397
	Ganzi	5	2	4	0.644 ± 0.152	0.00190	1.489	−0.63193
	Nyingchi	3	2	3	0.618 ± 0.104	0.00153	1.200	0.58729
	Diqing	1	1	2	0.514 ± 0.069	0.00066	0.514	1.37595
	Shannan	2	1	3	0.431 ± 0.087	0.00057	0.448	−0.24788
	Shigatse	1	1	2	0.571 ± 0.094	0.00073	0.571	1.44416
	All highland chickens	8	5	9	0.750 ± 0.018	0.0016	1.250	−0.27902

**Table 3:** Nucleotide divergence *(Dxy)* and net genetic distance *(Da)* among 12 populations.

	Muchuan	Emei	Jiuyuan	Black-Tianfu	White-Tianfu	Haiyan	Doilungdêqên	Ganzi	Nyingchi	Diqing	Shannan	Shigatse
Muchuan		0.258	0.235	**0.092**	0.144	0.174	0.181	0.198	0.232	0.14	0.134	0.104
Emei	0.092		0.184	0.258	0.214	0.226	0.211	0.338	0.239	**0.339**	0.193	0.214
Jiuyuan	0.128	0.013		0.233	0.162	0.195	0.181	0.314	0.257	0.308	0.162	0.184
Black-Tianfu	**−0.002**	0.099	0.135		0.139	0.171	0.18	0.191	0.235	0.131	0.162	0.101
White-Tianfu	0.020	0.025	0.034	0.023		0.169	0.166	0.234	0.234	0.198	0.129	0.122
Haiyan	0.028	0.016	0.044	0.033	0.001		0.177	0.263	0.215	0.237	0.138	0.141
Doilungdêqên	0.047	0.014	0.043	0.055	0.011	**0**		0.268	0.190	0.25	0.118	0.132
Ganzi	0.04	0.116	0.152	0.042	0.055	0.062	0.080		0.318	0.14	0.226	0.2
Nyingchi	0.095	0.038	0.116	0.107	0.076	0.035	0.022	0.126		0.319	0.15	0.175
Diqing	0.052	0.187	**0.216**	0.051	0.088	0.105	0.131	0.003	0.197		0.199	0.157
Shannan	0.051	0.045	0.074	0.074	0.024	0.011	0.004	0.088	0.032	0.13		**0.076**
Shigatse	0.012	0.058	0.088	0.017	0.009	0.005	0.009	0.053	0.049	0.079	0.004	

*Note*. Lower diagonal was nucleotide divergence *Da*; upper diagonal was net genetic distance *Dxy*; all the values were enlarged 100 times.

**Table 4:** Coefficient of differentiation *(Gst)* and gene flow *(Nm)* between populations.

	Muchuan	Emei	Jiuyuan	Black-Tianfu	White-Tianfu	Haiyan	Doilungdêqên	Ganzi	Nyingchi	Diqing	Shannan	Shigatse
Muchuan		0.65	0.37	**−29.34**	2.63	2.46	1.17	0.66	0.54	0.76	0.44	1.75
Emei	0.28^*∗*^		9.23	0.66	4.39	5.88	3.05	1.4	1.19	0.85	0.67	1.2
Jiuyuan	0.40^*∗*^	0.03^*∗*^		0.33	2.14	1.66	1.23	0.9	0.66	0.56	0.4	0.86
Black-Tianfu	**−0.01**	0.27^*∗*^	0.43^*∗*^		2.89	1.88	0.93	0.71	0.54	0.76	0.38	1.64
White-Tianfu	0.09^*∗*^	0.05	0.11^*∗*^	0.08^*∗*^		**25.21**	**4.9**	1.31	0.98	1.23	0.93	2.94
Haiyan	0.09^*∗*^	0.04	0.13^*∗*^	0.12^*∗*^	**0.01**		32.22	2.81	3.63	2.13	2.04	7.7
Doilungdêqên	0.18^*∗*^	0.08^*∗*^	0.17^*∗*^	0.21^*∗*^	**0.05**	0.01		2.07	6.55	1.46	5.45	13.33
Ganzi	0.28^*∗*^	0.15^*∗*^	0.22^*∗*^	0.26^*∗*^	0.16^*∗*^	0.08^*∗*^	0.11^*∗*^		1.01	16.95	0.76	1.06
Nyingchi	0.32^*∗*^	0.17^*∗*^	0.28^*∗*^	0.32^*∗*^	0.20^*∗*^	0.06^*∗*^	0.04	0.20^*∗*^		0.72	4.57	3.46
Diqing	0.25^*∗*^	0.23^*∗*^	0.31^*∗*^	0.25^*∗*^	0.17^*∗*^	0.11^*∗*^	0.15^*∗*^	0.02	0.26^*∗*^		0.59	1.12
Shannan	0.36^*∗*^	0.27^*∗*^	0.39^*∗*^	0.40^*∗*^	0.21^*∗*^	0.11^*∗*^	0.04	0.25^*∗*^	0.52^*∗*^	0.30^*∗*^		10.16
Shigatse	0.13^*∗*^	0.17^*∗*^	0.23^*∗*^	0.13^*∗*^	0.08^*∗*^	0.03	0.02	0.19	0.07	0.18^*∗*^	0.02	

*Note*. Lower diagonal was nucleotide divergence *Gst*, upper diagonal was net genetic distance *Nm*; ^*∗*^*P* < 0.05 meant significant difference between lowland chicken and Tibetan chicken breed.

**Table 5:** Analysis of molecular variance of *COX-III* sequences in 12 populations.

Source of variation	Sum of squares	df	Variance components	Percentage	Fixation index
Among populations	56.807	11	0.19 (Va)	29.57	0.2957^*∗∗*^
Within populations	132.731	288	0.46 (Vb)	70.43	

*Note*. Fixation index: 0.2957; ^*∗∗*^*P* < 0.01.

**Table 6:** Distribution of SNPs in *COX-III* gene in Tibetan chickens and lowland chickens.

SNP sites	Allele distribution			*P* value in Pearson chi-square test
	Allele	TC^a^	LC^b^	
10017	C	154 (99.4%)	145 (100%)	0.33263
	A	1 (0.6%)	0 (0%)	
10081	A	90 (58.1%)	62 (42.8%)	0.00805^*∗*^
	G	65 (41.9%)	83 (57.2%)	
10112	G	155 (100%)	144 (99.3%)	0.30037
	A	0 (0%)	1 (0.7%)	
10115	G	140 (90.3%)	145 (100%)	0.00012^*∗*^
	A	15 (9.7%)	0 (0%)	
10162	G	155 (100%)	144 (99.3%)	0.30037
	A	0 (0%)	1 (0.7%)	
10270	G	14 (9%)	136 (93.8%)	9.62 × 10^−49^^*∗*^
	A	141 (91.0%)	9 (6.2%)	
10336	A	155 (100%)	144 (99.3%)	0.30037
	G	0 (0%)	1 (0.7%)	
10369	G	154 (99.4%)	145 (100%)	0.33263
	A	1 (0.6%)	0 (0%)	
10555	G	154 (99.4%)	145 (100%)	0.33263
	A	1 (0.6%)	0 (0%)	
10587	T	21 (13.5%)	94 (64.8%)	6.92 × 10^−20^^*∗*^
	C	134 (86.5%)	51 (35.2%)	
10809	C	143 (92.3%)	136 (93.8%)	0.60255
	T	12 (7.7%)	9 (6.2%)	

Note: relative content in parentheses meant the number of birds in corresponding allele in the specific polymorphic site; ^*∗*^*P* < 0.05 meant significant difference between lowland chicken and Tibetan chicken breeds; ^a^TC was the abbreviations for Tibetan chicken; ^b^LC was the abbreviations for lowland chicken.

**Table 7:** Variable sites of* COX-III* haplotype sequences of chickens.

Haplotype reference sequence		Position of variable sites in *COX-III* gene										Number of birds in highland chickens (%)	Number of birds in lowland chickens (%)	Number of birds
	10017	10081	10112	10115	10162	10270	10336	10369	10555	10587	10809			
	C	A	G	G	G	G	A	G	G	T	C			
Ha1	**·**	G	**·**	**·**	**·**	**·**	**·**	**·**	**·**	**·**	**·**	49 (31.6%)	81 (55.9%)	130
Ha2	**·**	**·**	**·**	**·**	**·**	**·**	**·**	**·**	**·**	**·**	**·**	54 (34.8%)	2 (1.4%)	56
Ha3	**·**	G	**·**	A	**·**	**·**	**·**	**·**	**·**	**·**	**·**	15 (9.7%)	—	15
Ha4	**·**	**·**	**·**	**·**	**·**	**·**	**·**	**·**	**·**	**·**	**·**	1 (0.6%)	—	1
Ha5	**·**	**·**	**·**	**·**	**·**	**·**	**·**	**·**	**·**	**·**	**·**	21 (13.5%)	50 (34.5%)	71
Ha6	**·**	**·**	**·**	**·**	**·**	**·**	**·**	**·**	**·**	**·**	**·**	12 (7.7%)	9 (6.2%)	21
Ha7	**·**	**·**	**·**	**·**	**·**	**·**	**·**	**·**	**·**	**·**	**·**	1 (0.6%)	—	1
Ha8	**·**	G	**·**	**·**	**·**	**·**	**·**	**·**	A	C	**·**	1 (0.6%)	—	1
Ha9	**·**	G	**·**	**·**	**·**	**·**	G	**·**	**·**	**·**	**·**	—	1 (0.7%)	1
Ha10	A	**·**	**·**	**·**	**·**	**·**	**·**	**·**	**·**	**·**	**·**	1 (0.6%)	—	1
Ha11	**·**	**·**	A	**·**	**·**	**·**	**·**	**·**	**·**	**·**	**·**	—	1 (0.7%)	1
Ha12	**·**	G	**·**	**·**	**·**	**·**	**·**	**·**	**·**	**·**	**·**	—	1 (0.7%)	1
AA Subst	*Ser29Tyr*		*Val61Met*	*Val62Ile*					*Ala162Thr*					

*Note*. Black dot (**·**) indicates the same base as the reference, Ha1–Ha12 are haplotypes, and dashes (—) represented the absence of certain haplotype in the population amino acid substitutions (AA Subt.) that were listed below the nucleotide information and marked in italic; position of variable sites in the *COX-III* gene of chickens was obtained using the complete mitochondrial genome sequence of RJF as the reference sequence.

**Table 8:** Patterns of haplotype distribution in *COX-III* gene.

Haplotype	Number of Tibetan chickens	Number of lowland chickens	*P* value	OR	95% CIs
Ha1	49	81	0.00035^*∗*^	0.484	0.32515–0.72169
Ha2	54	2	1.911 × 10^−14^^*∗*^	30.375	7.332–125.837
Ha3	15	0	0.00006^*∗*^	—	—
Ha4	1	0	1.000	—	—
Ha5	21	50	0.00008^*∗*^	0.349	0.20373–0.59712
Ha6	12	9	0.66178	1.257	0.52175–3.030
Ha7	1	0	1.000	—	—
Ha8	1	0	1.000	—	—
Ha9	0	1	0.483	—	—
Ha10	1	0	1.000	—	—
Ha11	0	1	0.483	—	—
Ha12	0	1	0.483	—	—

Total	155	145			

Note: OR: odds ratio. OR < 1 mean haplotype may be negatively associated with high-altitude adaptation; OR = 1 mean haplotype is not associated with high-altitude adaptation; OR > 1, haplotype may be surely associated with high-altitude adaptation. CIs: confidence intervals; ^*∗*^*P* < 0.05 meant significant difference between lowland chicken and Tibetan chicken breeds.
